# Socio-economic factors, gender and smoking as determinants of COPD in a low-income country of sub-Saharan Africa: FRESH AIR Uganda

**DOI:** 10.1038/npjpcrm.2016.50

**Published:** 2016-09-01

**Authors:** Frederik van Gemert, Niels Chavannes, Bruce Kirenga, Rupert Jones, Sian Williams, Ioanna Tsiligianni, Judith Vonk, Janwillem Kocks, Corina de Jong, Thys van der Molen

**Affiliations:** 1 Department of General Practice, Groningen Institute for Asthma and COPD (GRIAC), University of Groningen, University Medical Center Groningen, Groningen, The Netherlands; 2 Department of Public Health and Primary Care, Leiden University Medical Center, Leiden, The Netherlands; 3 Division of Pulmonary Medicine, Makerere University Lung Institute, Mulago Hospital, Makerere University, Kampala, Uganda; 4 Centre for Clinical Trials and Population Studies, Plymouth University Peninsula School of Medicine and Dentistry, Plymouth, UK; 5 Executive Office, International Primary Care Respiratory Group, London, UK; 6 Department of Epidemiology, Groningen Research Institute for Asthma and COPD (GRIAC), University of Groningen, University Medical Center Groningen, Groningen, The Netherlands

## Abstract

In Uganda, biomass smoke seems to be the largest risk factor for the development of COPD, but socio-economic factors and gender may have a role. Therefore, more in-depth research is needed to understand the risk factors. The aim of this study was to investigate the impact of socio-economic factors and gender differences on the COPD prevalence in Uganda. The population comprised 588 randomly selected participants (>30 years) who previously completed the FRESH AIR Uganda study. In this *post hoc* analysis, the impact of several socio-economic characteristics, gender and smoking on the prevalence of COPD was assessed using a logistic regression model. The main risk factors associated with COPD were non-Bantu ethnicity (odds ratio (OR) 1.73, 95% confidence interval (CI) 1.06–2.82, *P*=0.030), biomass fuel use for heating (OR 1.76, 95% CI 1.03–3.00, *P*=0.038), former smoker (OR 1.87, 95% CI 0.97–3.60, *P*=0.063) and being unmarried (OR 0.087, 95% CI 0.93–2.95, *P*=0.087). A substantial difference in the prevalence of COPD was seen between the two ethnic groups: non-Bantu 20% and Bantu 12.9%. Additional analysis between these two groups showed significant differences in socio-economic circumstances: non-Bantu people smoked more (57.7% vs 10.7%), lived in tobacco-growing areas (72% vs 14.8%) and were less educated (28.5% vs 12.9% had no education). With regard to gender, men with COPD were unmarried (OR 3.09, 95% CI 1.25–7.61, *P*=0.015) and used more biomass fuel for heating (OR 2.15, 95% CI 1.02–4.54, *P*=0.045), and women with COPD were former smokers (OR 3.35, 95% CI 1.22–9.22, *P*=0.019). Only a few socio-economic factors (i.e., smoking, biomass fuel use for heating, marital status and non-Bantu ethnicity) have been found to be associated with COPD. This applied for gender differences as well (i.e., for men, marital status and biomass fuel for heating, and for women being a former smoker). More research is needed to clarify the complexity of the different risk factors.

## Introduction

Chronic obstructive pulmonary disease (COPD) is a major health problem in low- and middle-income countries (LMICs).^1^ In 2010, COPD was the fourth leading cause of death globally, and it was expected to be the third by 2030.^2,3^ Unfortunately, the prediction has been overtaken by reality: at this moment, COPD is the third leading cause of mortality worldwide.^4,5^ Approximately 90% of COPD deaths occur in LMICs.^6^ Despite these high numbers, COPD is an unknown disease in most of the rural areas of sub-Saharan Africa, both in terms of public awareness and in public health planning. The people are unaware of the potential damage to respiratory and non-respiratory health caused by tobacco and biomass smoke.^7–9^ Biomass fuel use is the third largest contributor to the global burden of disease.^10^

Although the development of COPD is multifactorial, biomass smoke is probably the largest risk factor for COPD in LMICs.^11–13^ Worldwide, around 3 billion people, most of them living in LMICs, rely on the use of open fires and burning of biomass fuels (wood, animal dung, crop residues, straw and charcoal) for cooking and heating in poorly ventilated conditions.^14^ Solid fuel burning is incomplete and produces high levels of household air pollution with a range of more than 250 health-damaging pollutants, including carbon monoxide, nitrogen and sulphur oxides, as well as a variety of pollutants, irritants, carcinogens, co-carcinogens and free radicals.^12,13,15^

Until recently, data on the prevalence of COPD, the risk factors and socio-economic determinants in LMICs were scarce.^9,16,17^ In 2012, a prospective cross-sectional observational study (FRESH AIR Uganda) was conducted to assess the prevalence of COPD and its risk factors in a rural district of Uganda. Among adults above the age of 30 years, the prevalence of spirometry-based COPD was 16.2% (52.6% women), as defined according to the methods used in FRESH AIR Uganda.^18^ The prevalence of COPD was remarkably high (39%) among adults aged 30–39 years, both for men (37%) and for women (40%). In addition to tobacco smoking, particularly by young men, >90% of the participants were exposed to smoke caused by biomass fuel use.^18^

The FRESH AIR Uganda study was conducted in rural Masindi district (population 350,000) of Uganda, a low-income country with an average life expectancy of 52 years (men 48 and women 57).^19^ Masindi district is one of the poorest districts of Uganda, where the poverty line ($1.25 a day) is consistently above 40%.^19^ Poverty is known to be a risk factor for COPD, but the socio-economic factors that contribute to this are unclear, particularly in LMICs.^3,20^ The socio-economic status (SES) is an important determinant of overall health status.^17^ In contrast to poverty, which is often quantified as a minimum level of income to meet the basic needs of life, SES is defined as an individual overall position or standing, and it can be indicated by a compilation of measurements including income, as well as education, employment, location of residence, cooking tradition, biomass fuel use and housing.^21^ Using the data of FRESH AIR Uganda, we performed a *post hoc* analysis to examine the association of socio-economic factors, gender and smoking with COPD.

## Results

### Patient characteristics

Of the 588 participants, 95 (16.2%) were classified as having spirometry-based COPD and 493 (83.8%) as non-COPD (Table 1). Wood was used as main domestic fuel by 558 (94.9%) participants; grass was used by 534 (91%) participants; and crop residues were used by 501 (85.2%) participants; they were applied to light the fire. With regard to their cooking place, 490 (83.3%) participants cooked in a separate building as kitchen.

Participants with COPD coughed and wheezed more than those without COPD, particularly among men (all *P* values<0.007). There were no significant differences in age and gender between participants with and without COPD. More than 90% of the participants had at least one chest infection a year. More details are depicted in Table 1.

### Socio-economic factors, ethnicity and tobacco smoking as determinants of COPD

Participants with COPD were more often active or former smokers compared with participants without COPD (*P*=0.046), they used more biomass fuel for heating (*P*=0.035) and they were more often of non-Bantu ethnicity (*P*=0.020); a trend was shown for being unmarried (*P*=0.055). No other significant differences in socio-economic factors were found between subjects with and without COPD (Table 2).

In the multivariable logistic regression model on the presence of COPD (Table 3), significant associations were found with biomass fuel use for heating (odds ratio (OR) 1.76, 95% confidence interval (CI) 1.03–3.00, *P*=0.038) and non-Bantu ethnicity (OR 1.73, 95% CI 1.06–2.82, *P*=0.030). Borderline significant associations were found with being unmarried (OR 1.66, 95% CI 0.93–2.95, *P*=0.087) and being a former smoker (OR 1.87, 95% CI 0.97–3.60, *P*=0.063). No significant associations between the presence of COPD and educational level, employment, village in tobacco-growing areas, time cooking indoors or outdoors, sleeping area and cooking area were found.

### Risk factors for COPD stratified by gender

The logistic regression models on the presence of COPD stratified by gender showed for men an association with being unmarried (OR 3.09, 95% CI 1.25–7.61, *P*=0.015) and biomass fuel use for heating (OR 2.15, 95% CI 1.02–4.54, *P*=0.045), and for women an association with being a former smoker (OR 3.35 (95% CI 1.22–9.22, *P*=0.019; Table 3)).

### Cooking tradition as COPD determinant

There were no significant differences between non-COPD and COPD participants concerning the cooking data, including exposure to biomass smoke (hours per day and number of years), and sleeping area. There were similarly no significant differences between cooking data as risk factors and COPD prevalence.

## Discussion

### Main findings

The main risk factors associated with the COPD found during the FRESH AIR survey were being of non-Bantu ethnicity and biomass fuel use for heating; a trend was found with former smokers and marital status. An association with biomass fuel use for cooking, both indoors and outdoors, was not found, as almost everybody used biomass fuel for cooking. The other tested socio-economic factors also did not differ between subjects with and without COPD. Among men, a risk factor for COPD was being unmarried and the use of biomass fuel for heating; a borderline risk factor was being of non-Bantu ethnicity. Among women, being a former smoker was a risk factor for COPD.

### Interpretation of findings in relation to previously published work

Participants with COPD were more often active or former smokers compared with participants without COPD and used more biomass fuel for heating (*P* values 0.046 and 0.035, respectively). This is well known from the literature.^3,12^ However, it was striking to see in the multivariable analysis that former smoking, and not active smoking, was borderline associated with a higher prevalence of COPD. This was particularly true for women, whereas in men no association was found at all. These findings were not confirmed by other studies. A possible explanation for this could be the so-called ‘healthy smoker effect’.^22^ This refers to the fact that people who quit smoking often do this motivated by smoking-related symptoms, leaving the less suffering and relatively healthy group still smoking. In our study, this was only seen in women. In Ugandan men, the decision to quit smoking is probably not related to health- or smoking-related symptoms, but it may be influenced by other factors (e.g., cultural factors) that need to be discovered.

In addition, tobacco smoke potentiates the detrimental effects of biomass smoke;^11,23,24^ active smokers, who are also exposed to biomass smoke, have an increasing risk of airflow obstruction.^25,26^ Given that almost every participant in our study was exposed to biomass fuel used for cooking, this variable could not be investigated. However, the use of biomass fuel for heating was significantly associated with the prevalence of COPD, especially in men. This indicates that biomass fuel use is indeed an important risk factor for COPD.

For men, being unmarried seemed to increase the risk of developing COPD. This could be explained by the fact that married men are less exposed to biomass fuel for cooking, as women in Uganda, and probably other countries of sub-Saharan Africa, have the responsibility for domestic cooking.^7,27^ Although women were more exposed to biomass smoke (both number of years and hours per day), no association with COPD was found. However, during cooking, women and perhaps unmarried men have several periods of intense exposure to biomass smoke each day, particularly when fires are started or stirred.^7,27^ More research is needed to understand the individual exposure to household air pollution, as the exposure is spatially and temporally highly variable.^28^ For men, the biomass fuel exposure for heating increased the chance of COPD: the context of this finding is not clear yet.

A substantial difference in the prevalence of COPD was seen between the two ethnic groups: the prevalence of COPD among non-Bantu people was 20% (20.3% men and 19.7% women) and among Bantu people it was 12.9% (10.5% men and 14.9% women). Interestingly, additional analyses showed substantial differences between the two ethnic groups in SES. Bantu refers to a primarily large and complex linguistic grouping of people in Africa. Their cultural pattern is extremely diverse and are the most prosperous. They occupy the southern and western parts of Uganda.^19,29^ In general, non-Bantu people are the poorer ethnic group, and they inhabit a geographical area stretching semi-arid eastern and northern parts of Uganda.^19,29^ Compared with the Bantu people, non-Bantu smoked more (57.7% vs 10.7%, *P*<0.001)), were less educated (no education 28.5% vs 12.9%, particularly women: 51.6% vs 17.1%, *P*<0.001) and lived more in tobacco-growing areas (72.0% vs 14.8%, *P*<0.001). After adjustment for these socio-economic factors in the multivariable model, the association between ethnicity and COPD remained significant, in contrast to the single socio-economic risk factors (tobacco smoking, education and living in tobacco-growing areas). An explanation for this could be that ethnicity was associated with a combination of all these socio-economic factors, and that this combination was more important than any single factor. As such, ethnicity could be seen as a variable indicating SES. However, other unmeasured factors, such as lifestyle, cultural or genetic factors, that differ between the ethnic groups could also explain this association between COPD and ethnicity. Further research is necessary to confirm this.

### Strengths and limitations of this study

FRESH AIR Uganda was one of the first observational surveys on the prevalence of COPD performed in a rural area of sub-Saharan Africa. The survey used well-trained local healthcare workers and was performed in 30 villages, randomly selected with a probability proportional to their size.^18^ The sample size was relatively small, but had enough power to detect differences in COPD prevalence between men and women in Masindi district. However, it was not powered to detect significant differences in COPD prevalence among other sub-groups (e.g., ethnicity or occupational groups). In addition, it was not possible to detect a difference in COPD prevalence associated with exposure to biomass smoke, as the exposure was almost uniform in this rural area.^18^ Finally, this study was a *post hoc* analysis, and the results need caution with interpretation. Further, properly designed prospective studies are needed to confirm our findings.

### Implications for future research, policy and practice

Tobacco smoking is known to be a major cause of COPD, but recent literature has shown that the use of biomass fuels for cooking and heating is an important risk factor as well, particularly in LMICs.^12,13^ A person living in a rural area of sub-Saharan Africa is exposed to a variety of other risk factors for the development of COPD during all stages of life: perinatal factors (maternal exposure to biomass smoke or tobacco smoke, low birth weight and pre-term birth), childhood exposure (respiratory tract infections, exposure to biomass smoke, childhood asthma, second-hand smoking, occupational exposure, poor nutrition and kerosene-based lamps) and adult exposure (occupational exposure, agricultural smoke, exposure to biomass smoke, cigarette smoking, second-hand smoking, kerosene-based lamps and outdoor air pollution).^30–32^ More information is needed to understand the full extent and influence of these risk factors. Low socio-economic circumstances such as poverty are associated with most of these risk factors, as well as poor access to healthcare, poor living conditions and water supply/sanitation. All these factors may cause health effects (intrauterine growth restriction, malnutrition, respiratory tract infections) and therefore increase the risk of developing COPD.^3,17,20,33–35^ The influence of socio-economic factors is therefore very complex: more research is needed to identify these partly modifiable risk factors on the development of COPD.^17,34,35^

The general lack of knowledge leads to failure to make simple steps in avoiding exposure to biomass smoke.^30,36^ The nature of the communities also determines the health-seeking behaviour, both traditional (local herbs) and western (dispensaries and health centres), most of the time with a lack of successful results and not addressing the problem of exposure.^7^ Reduction of tobacco smoking and exposure of biomass smoke, as well as second-hand tobacco smoke, smoke from kerosene lamps and occupational air pollution, are major controllable factors to tackle the burden of COPD. Public awareness and control of (household) environment are important steps in preventing respiratory and non-respiratory diseases.^36^ More research is vital, with prospective studies and a larger sample size, to perform further comparisons among the sub-groups, and to understand the impact of COPD and other (non)-respiratory diseases.

### Conclusions

The risk factors for the development of COPD in Masindi district of Uganda are complex. Although tobacco smoking remains an important cause of COPD, almost everybody in this district, and probably in many other rural areas of sub-Saharan Africa, is exposed to biomass smoke and other risk factors. Only a few socio-economic factors have been found to be significantly associated with COPD (biomass fuel use for heating and non-Bantu ethnicity); for others, a trend was found (former smoker and marital status). This applied for gender differences as well (i.e., marital status and biomass fuel for heating for men, and former smoker for women). Between the two ethnic groups of Masindi district in Uganda, a difference in the prevalence of COPD was found, which could possibly be explained by the combination of several unfavourable socio-economic circumstances in the non-Bantu people. Research is needed to elucidate the complexity of the different risk factors in the development of COPD. Any intervention to reduce the incidence of COPD must combine raising awareness about the damaging effects of biomass fuel use and tobacco smoking, with clean-cooking solutions and tobacco smoking cessation to support at-risk communities. Researchers, policymakers and government, stakeholders, health professionals and communities will have to work together to control the growing burden of COPD, and start prevention and intervention programmes.

## Materials and methods

### The FRESH AIR Uganda study

The intended sample size of the FRESH AIR Uganda study was 600 participants, determined to give an acceptable degree of reliability in estimating the prevalence of spirometry-based COPD.^37^ Eventually, 588 randomly selected participants were asked about their living circumstances and exposure to risk factors.^18^ A screening questionnaire assessed gender, tribal and ethnic origin, education, living conditions, occupation, biomass fuel use, tobacco smoking, symptoms, MRC dyspnoea score and chest infections.^18^ A household air pollution questionnaire gave information on type and place of cook stoves, type of fuels, preparation of meals, time activity pattern and cooking during pregnancy. Both questionnaires were developed from different validated questionnaires, and were pre-tested and completed during a face-to-face interview.^18^ Subsequently, to assess COPD prevalence, pre- and post-bronchodilator spirometry was performed by well-trained local healthcare workers.^18^ The study used the lower limit of normal threshold—i.e., participants below the fifth percentile of the predicted FEV_1_/FVC ratio—as the defining criterion of COPD to avoid under-diagnosis in young participants and over-diagnosis in older participants.^38,39^

The study was approved by the Makerere University School of Medicine Ethics Committee and the Uganda National Council for Science and Technology (HS 2012-1142). All participants signed an informed consent form, or in case of illiteracy thumb-printed and signed by the village leader. More details about the study participants, study procedure and COPD diagnosis of FRESH AIR Uganda are reported elsewhere.^18^

### Socio-economic status, ethnicity and tobacco smoking

FRESH AIR Uganda measured the SES using education, employment, location of residence, exposure to household air pollution, cooking tradition, sleeping and cooking areas as variables. Furthermore, we also included variables such as gender, ethnicity and tobacco smoking to capture possible inequalities within communities. Ethnicity was defined as Bantu speaking versus non-Bantu speaking based on linguistic grouping of the 55 tribes living in Masindi district.^29,40,41^

### Statistical methods

Demographic and socio-economic characteristics of subjects with and without COPD were compared using chi-square tests for categorical variables, Student’s *t*-test for normally distributed continuous variables and Mann–Whitney *U*-tests for non-normally distributed variables.

The association between socio-economic factors and COPD was assessed using a multivariable logistic regression model adjusted for age, gender and smoking habits. Socio-economic factors were selected using backward selection, and the final model retained all socio-economic variables with a *P* value <0.1. The socio-economic factors tested were marital status, biomass fuel use for cooking (indoors and outdoors), biomass fuel use for heating, ethnicity, education, employment, village in tobacco-growing area and sleeping area. The analyses were also stratified for gender to assess risk factors for COPD in men and women separately. *P* values <0.05 were considered statistically significant, and *P* values of 0.05–0.10 were considered trends. We performed the statistical analyses with the Statistical Package for the Social Science SPSS 20 (IBM SPSS, New York, USA).

## Funding

The International Primary Care Respiratory Group received an unrestricted grant from Mundipharma International to conduct the FRESH AIR Uganda survey.

## Figures and Tables

**Table 1:** Comparison of symptoms between non-COPD and COPD participants and within gender

	*non-COPD*			*COPD*			P *value*		
	*Total*	*Men*	*Women*	*Total*	*Men*	*Women*	*Total*	*Men*	*Women*
Population	493 (83.8%)	246 (49.9%)	247 (50.1%)	95 (16.2%)	45 (47.4%)	50 (52.6%)			
Age	44.9 (13.5)	44.9 (13.1)	44.9 (14.0)	46.6 (13.9)	45.5 (11.5)	47.7 (15.5)	0.258	0.793	0.211
Cough	89 (18.1%)	40 (16.3%)	49 (19.8%)	29 (30.5%)	15 (33.3%)	14 (28.0%)	0.005	0.007	0.198
Phlegm	102 (20.7%)	48 (19.5%)	54 (21.9%)	21 (22.1%)	13 (28.9%)	8 (16.0%)	0.756	0.155	0.352
Wheeze	32 (6.5%)	16 (6.5%)	16 (6.5%)	16 (16.8%)	9 (20.0%)	7 (14.0%)	0.001	0.003	0.070
									
Chest infections							0.381	0.007	0.541
None	50 (10.1%)	30 (12.2%)	20 (8.1%)	9 (9.5%)	4 (8.9%)	5 (10.0%)			
1–2 per year	272 (55.2%)	134 (54.5%)	138 (55.9%)	46 (48.4%)	15 (33.3%)	31 (62.0%)			
>2 per year	171 (34.7%)	82 (33.3%)	89 (36.0%)	40 (42.1%)	26 (57.8%)	14 (28.0%)			

Data are *N* (%) or mean (s.d.).

Abbreviation: COPD, chronic obstructive pulmonary disease.

**Table 2:** Comparison of socio-economic and risk factors between non-COPD and COPD participants

	*Total*	*non-COPD*	*COPD*	P* value*
*Gender*				0.652
Men	291 (49.5%)	246 (49.9%)	45 (47.4%)	
Women	297 (50.5%)	247 (50.1%)	50 (52.6%)	
				
*Unmarried*				0.055
Yes	113 (19.2%)	88 (17.8%)	25 (26.3%)	
				
*Biomass fuel use indoors*				0.335
Yes	546 (92.9%)	460 (93.3%)	86 (90.5%)	
				
*Biomass fuel use outdoors*				0.704
Yes	544 (92.5%)	457 (92.7%)	87 (91.6%)	
				
*Biomass fuel for heating*				0.035
Yes	104 (17.7%)	80 (16.2%)	24 (25.5%)	
				
*Smoking*				0.046
Active	122 (20.7%)	98 (19.9%)	24 (25.3%)	
Former	87 (14.8%)	67 (13.6%)	20 (21.1%)	
None	379 (64.5%)	328 (66.5%)	51 (53.7%)	
				
*Ethnicity*				0.020
Non-Bantu	270 (45.9%)	216 (43.8%)	54 (56.8%)	
Bantu	318 (54.1%)	277 (56.2%)	41 (43.2%)	
				
*Education*				0.433
None	118 (20.1%)	97 (19.7%)	21 (22.1%)	
Primary	358 (60.9%)	302 (61.3%)	56 (58.9%)	
Secondary	91 (15.5%)	74 (15.0%)	17 (17.9%)	
Tertiary	21 (3.6%)	20 (4.1%)	1 (1.1%)	
				
*Employment*				0.585
Farmers	441 (75.0%)	370 (75.1%)	71 (74.7%)	
Business	43 (7.3%)	37 (7.5%)	6 (6.3%)	
Teachers	16 (2.7%)	15 (3.0%)	1 (1.1%)	
Others	69 (11.7%)	57 (11.6%)	12 (12.6%)	
Unemployed	19 (3.2%)	14 (2.8%)	5 (5.3%)	
				
*Village tobacco-growing area*				0.249
Yes	241 (41.0%)	197 (40.0%)	44 (46.3%)	
				
*Sleeping area*				0.711
Same room as kitchen	44 (7.5%)	35 (7.1%)	9 (9.5%)	
Separate room	54 (9.2%)	45 (9.1%)	9 (9.5%)	
Separate house	490 (83.3%)	413 (83.8%)	77 (81.1%)	

Data are *N* (%).

Abbreviation: COPD, chronic obstructive pulmonary disease.

**Table 3:** Results of multivariable analysis of risk factors for COPD for all subjects and stratified by gender

	*All subjects*		*Men*		*Women*	
	*OR (95% CI)*	P *value*	*OR (95% CI)*	P *value*	*OR (95% CI)*	P *value*
Age (years)	1.01 (0.99–1.02)	0.485	1.01 (0.98–1.04)	0.559	1.00 (0.98–1.03)	0.886

*Gender*						
Women	1 (reference)					
Men	0.74 (0.44–1.26)	0.269				
						
*Unmarried*						
No	1 (reference)		1 (reference)			
Yes	1.66 (0.93–2.95)	0.087	3.09 (1.25–7.61)	0.015		
						
*Biomass fuel for heating*						
No	1 (reference)		1 (reference)			
Yes	1.76 (1.03–3.00)	0.038	2.15 (1.02–4.54)	0.045		
						
*Smoking*						
Never	1 (reference)		1 (reference)		1 (reference)	
Active	1.57 (0.84–2.92)	0.156	1.53 (0.68–3.45)	0.310	1.27 (0.40–4.04)	0.692
Former	1.87 (0.97–3.60)	0.063	1.51 (0.61–3.72)	0.375	3.35 (1.22–9.22)	0.019
						
*Ethnicity*						
Bantu	1 (reference)		1 (reference)			
non-Bantu	1.73 (1.06–2.82)	0.030	1.94 (0.94–4.03)	0.075		

Abbreviations: CI, confidence interval; OR, odds ratio.

## Notes

N.C., I.T. and J.K. are Associate Editors of *npj Primary Care Respiratory Medicine*, but were not involved in the editorial review of, nor the decision to publish this article. The other authors declare no conflict of interest.
